# Editorial: Insights in biofabrication 2023: novel developments, current challenges, and future perspectives

**DOI:** 10.3389/fbioe.2025.1683662

**Published:** 2025-09-09

**Authors:** Sandra Van Vlierberghe, Izabela-Cristina Stancu

**Affiliations:** ^1^ Polymer Chemistry and Biomaterials Group, Centre of Macromolecular Chemistry, Department of Organic and Macromolecular Chemistry, Ghent University, Ghent, Belgium; ^2^ Advanced Polymer Materials Group, Faculty of Medical Engineering, National University of Science and Technology Politehnica of Bucharest, Bucharest, Romania

**Keywords:** bioprinting, bone tissue, liver lobule models, silk spinning, lung cancer treatment, microfluidics, holistic bioprinting

## Introduction

The landscape of biomedical research is rapidly transforming due to innovations in 3D bioprinting, bioinspired materials, and microfluidic technologies. Five recent articles from this Research Topic explore key advances in this dynamic field, spanning tissue regeneration, organ modeling, personalized medicine, and the democratization of bioprinting. Together, they paint a compelling picture of where biomedical engineering stands today and where it must go to address clinical and technological gaps.

## Engineering bone tissue through bioprinting: mimicking complexity

Bone regeneration has long been a formidable challenge due to the intricate hierarchical organization, architecture, and mechanobiology of native tissue. One article delves into how bioprinting is enabling spatially organized cellular niches that can replicate the bone’s functional complexity. The review categorizes approaches based on deposition methods—light-based versus extrusion—and crosslinking chemistries, specifically contrasting chain-growth systems like GelMA with step-growth systems such as thiol-ene, which hold promise for improved cell viability and encapsulation essential for bone regeneration.

Crucially, this work emphasizes that osteogenesis induced by bioprinted constructs depends on a synergistic interplay among the bioink’s biophysical cues, the printing method, and the cellular inputs. Moreover, the authors advocate for combining multiple bioprinting strategies to build hierarchical constructs with vascular and neural integration—a key to achieving long-term functionality. This multidimensional approach hints at the field’s future: not merely printing bone but recapitulating the developmental biology that builds it.

## Liver lobule models: precision through bioprinting and microfluidics

In another review, the liver is positioned as both a biological marvel and a tissue engineering challenge. The liver lobule, its fundamental unit, houses regionally specialized hepatocytes performing distinct functions. To reproduce this spatial and functional complexity *in vitro*, the authors highlight a dual-technology approach: 3D bioprinting for architectural precision and microfluidics for dynamic function.

These hybrid models represent a step forward for disease modeling, drug testing, and regenerative medicine, particularly for liver diseases that are poorly replicated by 2D cultures. The integration of microfluidics allows precise control over nutrient gradients and flow, better emulating the *in vivo* hepatic environment. This synthesis of technologies underscores a central theme in the field: biological realism requires technological convergence.

## Learning from nature: silk spinning as a model for sustainable polymers

Stepping outside the traditional tissue engineering paradigm, one review explores silk spinning as a bioinspired model for sustainable and high-performance polymer fabrication. Spiders and silkworms produce silk through a system of ambient, aqueous, and zero-waste processing, which stands in stark contrast to industrial polymer manufacturing.

The article focuses on solvent cues—pH, salt ions, and water content—that direct silk’s hierarchical assembly. These insights are critical for recreating silk spinning *in vitro*, which could enable a new generation of biocompatible, tunable, and recyclable materials for bioinspired polymer fabrication for medical devices. Although the underlying mechanisms remain incompletely understood, this area opens promising avenues for ecologically responsible biofabrication.

## Personalized lung cancer treatment: organoids meet microfluidics

Cancer treatment is moving rapidly toward personalization, and patient-derived organoids (PDOs) are emerging as pivotal tools. In the context of lung cancer, the review explores how PDOs replicate patient-specific tumor microenvironments and genetic heterogeneity. However, limitations such as low throughput and simplified tumor ecosystems restrict their translational utility.

The proposed solution lies in organoids-on-a-chip, merging PDOs with microfluidic platforms. This union enables real-time monitoring, dynamic flow control, and multi-tissue integration, potentially revolutionizing drug screening and therapy customization. Such tools are not only research breakthroughs—they are bridges toward precision oncology that adapts in real-time to the complexities of individual patients.

## Democratizing bioprinting: the promise of low-cost innovation

While much of the field pushes technical boundaries, cost remains a major barrier to global accessibility. One report tackles this challenge head-on by introducing a low-cost 3D bioprinter built from recycled materials and off-the-shelf electronics. Most commercial bioprinters range from $13,000 to $300,000, and bioinks can cost up to $100,000 per gram. By contrast, this prototype aims to dramatically reduce costs without compromising basic functionality.

This initiative represents more than engineering ingenuity—it’s a democratizing force in biomedical research. By making bioprinting accessible to resource-limited settings and educational institutions, it ensures a more inclusive future where innovation is not limited by geography or funding.

## Toward a holistic bioprinting paradigm

Taken together, these five articles chart a course toward a holistic, scalable, and accessible future for tissue engineering. The field is evolving from merely reproducing tissue shapes to replicating cellular function, vascular complexity, and dynamic responsiveness. Whether through mimicking bone mechanobiology, modeling liver and lung diseases with high fidelity, learning from nature’s polymer systems, or making bioprinting affordable for all, each article underscores a critical insight: the future of medicine lies at the intersection of biology, engineering, and accessibility.

But challenges remain. Cross-disciplinary integration, regulatory frameworks, and translational pathways must keep pace with technical innovations. Only then can bioprinting fulfill its promise—not just in labs and clinics but across the world.

